# Nuclear lipid droplets identified by electron microscopy of serial sections

**DOI:** 10.1186/1756-0500-6-386

**Published:** 2013-09-27

**Authors:** Rustem Uzbekov, Philippe Roingeard

**Affiliations:** 1 Plateforme des Microscopies, Université François Rabelais & CHRU de Tours, Tours, France; 2 Laboratoire de Biologie Cellulaire, INSERM U966, Faculté de Médecine, Université François Rabelais de Tours, 10 boulevard Tonnellé, Tours Cedex, 37032, France

**Keywords:** Lipid droplet, Nucleus, Electron microscopy, EM tomography

## Abstract

**Background:**

Recent studies have suggested that nuclear lipid droplets (LDs) are organized into domains similar to those of cytoplasmic LDs. As cytoplasmic LDs are formed at the endoplasmic reticulum (ER) membrane, which is structurally continuous with the nuclear envelope, it could be suggested however that nuclear LDs are cytoplamic LDs trapped within an invagination of the nuclear envelope. The resolution of fluorescence confocal microscopy is not sufficiently high to exclude this hypothesis.

**Findings:**

We therefore addressed this question by electron microscopy (EM) of serial sections. In human liver tissue, we observed some cytoplamic LDs partly surrounded by the nuclear compartment, but we were also able to identify LDs residing in the nuclear compartment that were not connected to the nuclear envelope.

**Conclusion:**

These findings indicate that nuclear LDs constitute specific subdomains of the nuclear compartment probably involved in nuclear lipid homeostasis.

## Findings

### Background

Cytoplasmic lipid droplets (LDs) are neutral lipid deposits present in almost all kinds of cell. They were first detected in cells by classical lipid staining of histological sections many years ago, but LDs have only recently begun to be regarded as independent organelles [1]. Unlike vesicular organelles, the aqueous contents of which are enclosed by a phospholipid bilayer, LDs consist of a highly hydrophobic lipid core surrounded by a phospholipid monolayer [2]. The prevailing model for LD formation involves the emergence of LDs from the ER lipid bilayer as a lens-shaped mass of neutral lipids that then buds off from the cytoplasmic face of the bilayer to form a droplet within the cytoplasm [3,4]. LDs have recently been promoted from the status of largely ignored static cytoplasmic inclusions to actively studied dynamic organelles regulating not only cellular lipid storage, but also lipid exchange with various organelles [5]. A meticulous analysis of the neutral lipids present in the nuclear compartment of cultured human hepatoma cells and rat liver recently suggested that nuclear lipids are not restricted to the nuclear membrane, with some lipids being organized in the nuclear matrix into domains similar to those of cytosolic LDs [6].

This has led to suggestions that nuclear LDs may represent a source of rapidly available fatty acids for nuclear membranes [6]. Cytoplasmic LDs are formed at the endoplasmic reticulum (ER) membrane [5], which is continuous with the nuclear envelope. It could be therefore suggested that nuclear LDs are actually cytoplasmic LDs trapped within an invagination of the nuclear envelope. The authors of the study concerned suggested that nuclear LDs must constitute a domain different from that created by nuclear envelope invagination, because they were resistant to detergents used in the isolation of nuclear matrix. However, they were unable to resolve this issue satisfactorily because the resolution of the fluorescence confocal microscopy technique they used was not high enough to exclude this possibility entirely [6]. We recently described the use of serial electron microscopy (EM) sections for the three-dimensional (3D) reconstruction of cultured cells accumulating cytoplasmic LDs following hepatitis C virus (HCV) infection [7]. We used a similar approach here, to analyze the spatial distribution of potential nuclear LDs present in the liver tissue of a patient chronically infected with HCV [8].

### Results and discussion

**Figure 1:**
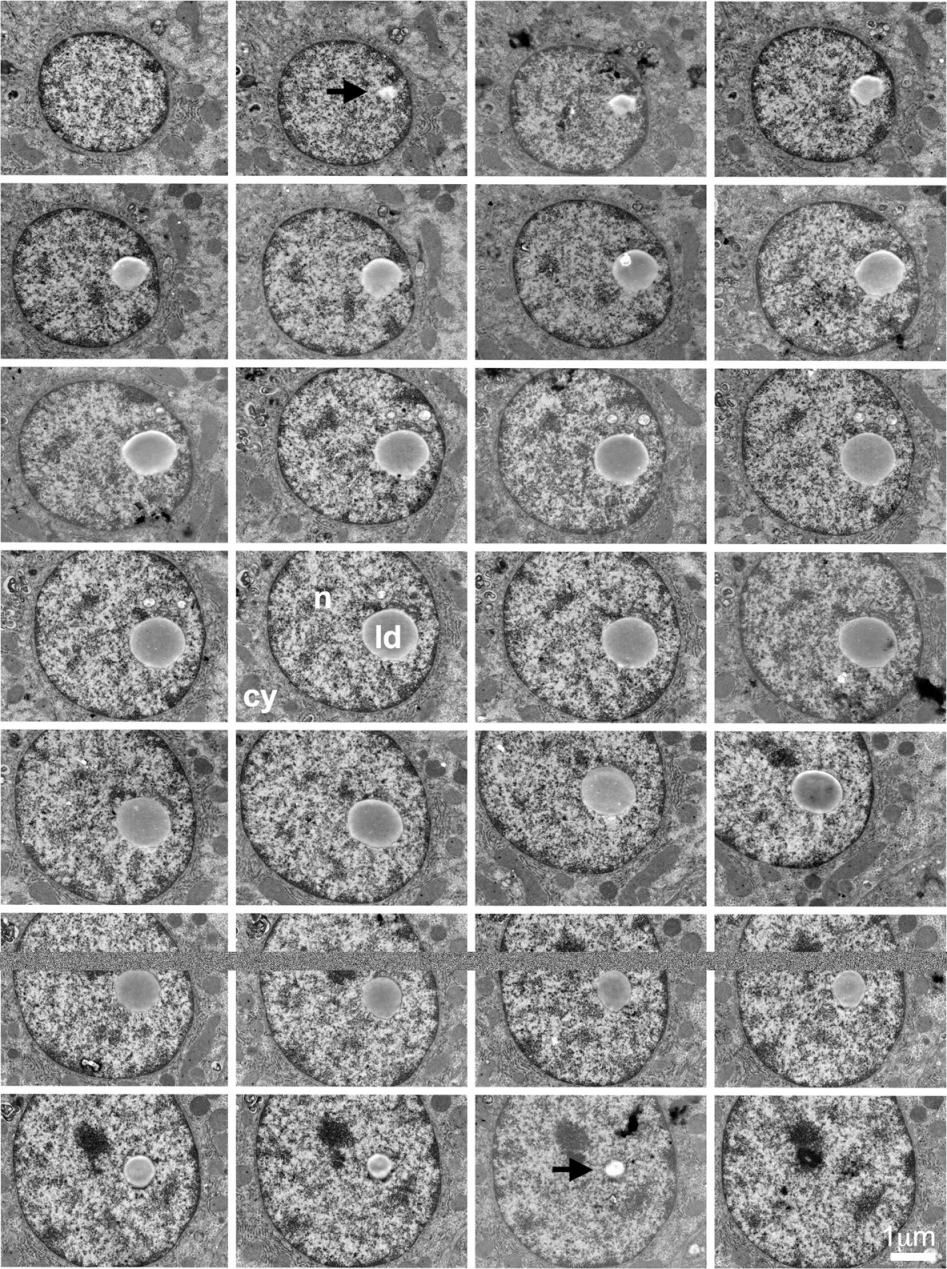
**Serial EM sections of liver tissue showing the presence of a nuclear lipid droplet.** None of these 30 consecutive serial sections showed any contact between the LD and the nuclear envelope, demonstrating the occurrence of an independent nuclear LD. Arrows indicate the LD in the first and last sections of the series in which it was possible to visualize the LD. ld: lipid droplet; n: nucleus; cy: cytoplasm.

**Figure 2:**
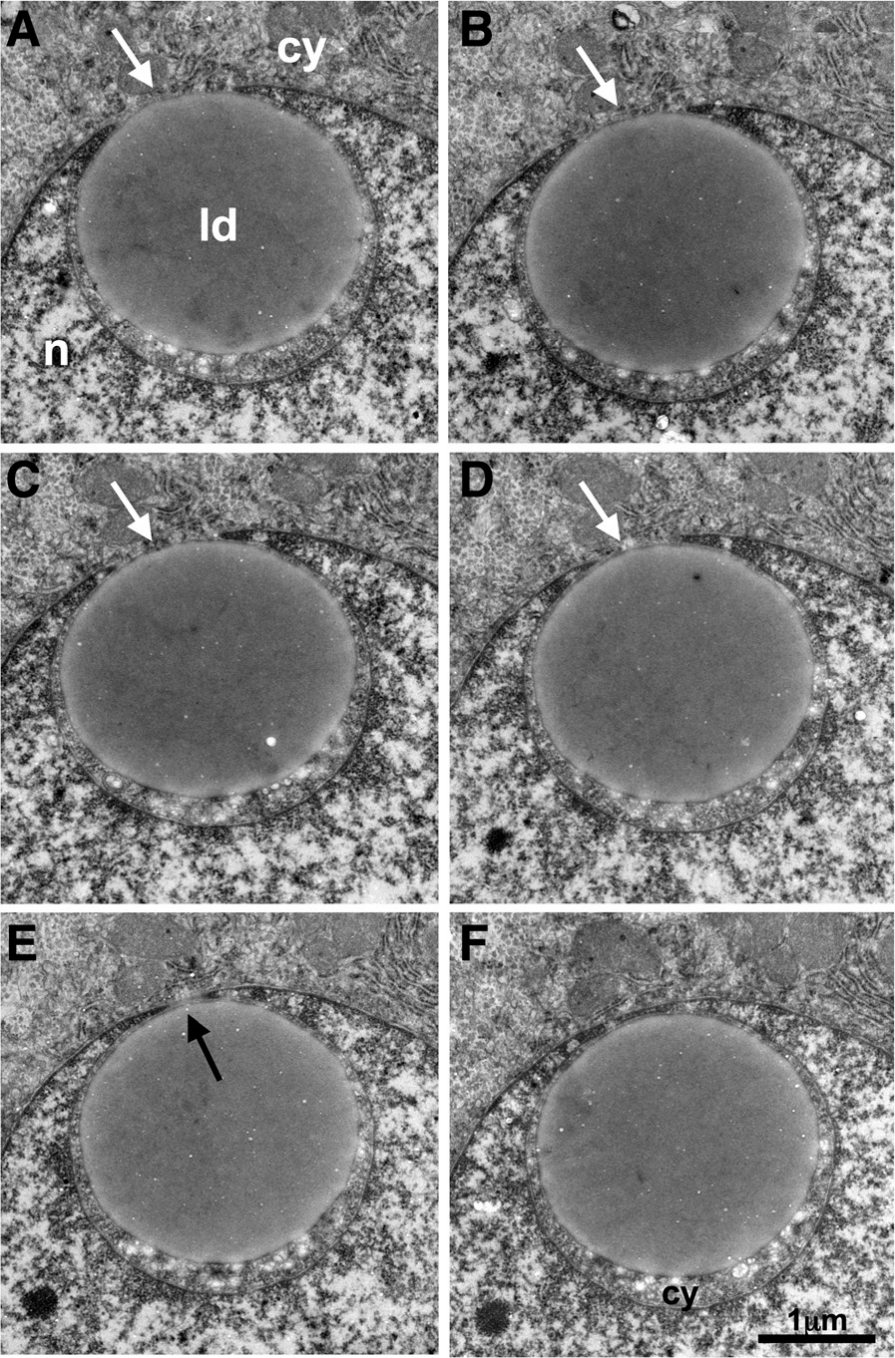
**Serial EM sections of liver tissue showing the presence of a cytoplasmic lipid droplet surrounded by the nucleus.** This series of 6 consecutive sections is a sample of the whole series (the whole stack consisted of 56 sections). The white arrows in **A**, **B**, **C** and **D** show contact between the LD and the cytoplasm. The black arrow in **E** indicates that the LD is also in contact with the nuclear envelope in this particular section. In electron micrograph in **F**, the LD appears to be completely surrounded by the nucleus, with a small portion of cytoplasm between the LD and the nuclear envelope. ld: lipid droplet; n: nucleus; cy: cytoplasm.

Figure 1 demonstrates unequivocally that truly nuclear LDs may be encountered in human liver tissues, as our analysis of consecutive serial EM sections covering the whole LD shown in this figure demonstrated that it was not connected to the nuclear envelope. Additional file 1: Figure S1 shows another example of a nuclear LD observed by a similar, EM tomography approach. By contrast, Figure 2 shows that some cytoplasmic LDs may be closely associated with the nucleus, even being partly surrounded by the nuclear compartment. It remains unclear whether this phenomenon reflects the translocation of a cytoplasmic LD into the nucleus, but this will be investigated in future investigations with cultured cell models. To appreciate the global percentage of these different types of LD, we performed the analysis of 583 hepatocytes from this liver biopsy. Among these cells, 117 (20%) did not contain any LD. Among the 466 hepatocytes that contained at least one LD, 402 (86%) had only isolated cytoplasmic LDs, while 60 (13%) presented a nucleus deformed by a large cytoplasmic LD or one LD partly surrounded by the nuclear compartment, and 4 (0.9%) presented at least one truly nuclear LD, not connected to the nuclear envelope. The observation of the different nuclear LDs by serial EM sections showed various degree of heterochromatin association. In some cases, the heterochromatin was present around the whole LD and visible in all serial sections (as in Additional file 1: Figure S1) while in other cases, only a partial association was visible in some sections of the series (as in Figure 1).

Nevertheless, our EM investigations with serial sections provide a more precise subcellular localization of LDs than confocal microscopy and confirmed the recently reported findings of Layenrenza *et al.*[6], suggesting that nuclear LDs may constitute a new class of subnuclear bodies. The role of these nuclear LDs found in about 1% of cells at a given time remains to be determined, but they may be directly involved in regulating nuclear-lipid metabolism, signaling events and interactions with transcription factors, such as PPAR and HNF 4α which have fatty acids as ligands [9]. However, our results do demonstrate that nuclear LDs constitute specific subdomains within the nucleus that store nuclear lipids and may be specifically involved in nuclear lipid homeostasis.

### Methods

Patient P03, a 47-year-old man, was previously included in a bioclinical study quantifying liver steatosis by monitoring LD number and size in the liver tissues of chronic carriers of HCV [8]. This patient was infected with a HCV 1b genotype and was classified as having steatosis of intermediate severity, characterized by a median cumulative LD area of 275 μm^2^ per 5000 μm^2^ of liver tissue [8]. Routine histological diagnosis demonstrated a minimally active chronic hepatitis graded A1-F1 with the METAVIR score. This patient, like all the other patients included in this previous study, signed a written informed consent form, in accordance with French regulations [8]. For EM analysis, the liver biopsy specimen was fixed by incubation for 48 h in 4% paraformaldehyde and 1% glutaraldehyde in 0.1 M phosphate buffer (pH 7.2) and postfixed by incubation for 1 h with 2% osmium tetroxide (Electron Microscopy Science, Hatfield, PA, USA). Liver tissue was then dehydrated by immersion in a graded series of ethanol solutions, cleared in propylene oxide, and embedded in Epon resin (Sigma), which was allowed to polymerize for 48 h at 60°C. Epon blocks were resized for the cutting of a thin ribbon of serial ultrathin sections (70 nm thick) of liver tissue. This ribbon was placed on EM grids, stained with 1% uranyl acetate and observed with a Jeol 1011 electron microscope (Tokyo, Japan). Electron micrographs were collected with a Gatan digital camera driven by Digital Micrograph software (Gatan, Plesanton, CA, USA) making it possible to visualize the same region of tissue in each of the series of sections.

## Abbreviations

EM: Electron microscopy; ER: Endoplasmic reticulum; HCV: Hepatitis C virus; LD: Lipid droplet.

## Competing interests

The authors declare that they have no competing interests.

## Authors’ contributions

RU and PR designed and performed the study, analyzed the data and wrote the manuscript. Both authors read and approved the final manuscript.

## Supplementary Material

Additional file 1: Figure S1 Serial EM sections of liver tissue showing the presence of a nuclear lipid droplet. These 18 consecutive serial sections show the presence of a nuclear LD, not connected to the nuclear envelope. Arrows indicate the LD in the first and the last sections of the series in which it was possible to visualize the LD. ld: lipid droplet; n: nucleus; cy: cytoplasm. Click here for file
