# Supplementary figures and images for: Nuclear lipid droplets identified by electron microscopy of serial sections
Source: BMC Res Notes. 2013 Sep 27;6:386. doi: 10.1186/1756-0500-6-386 (PMC3849021; doi:10.1186/1756-0500-6-386)

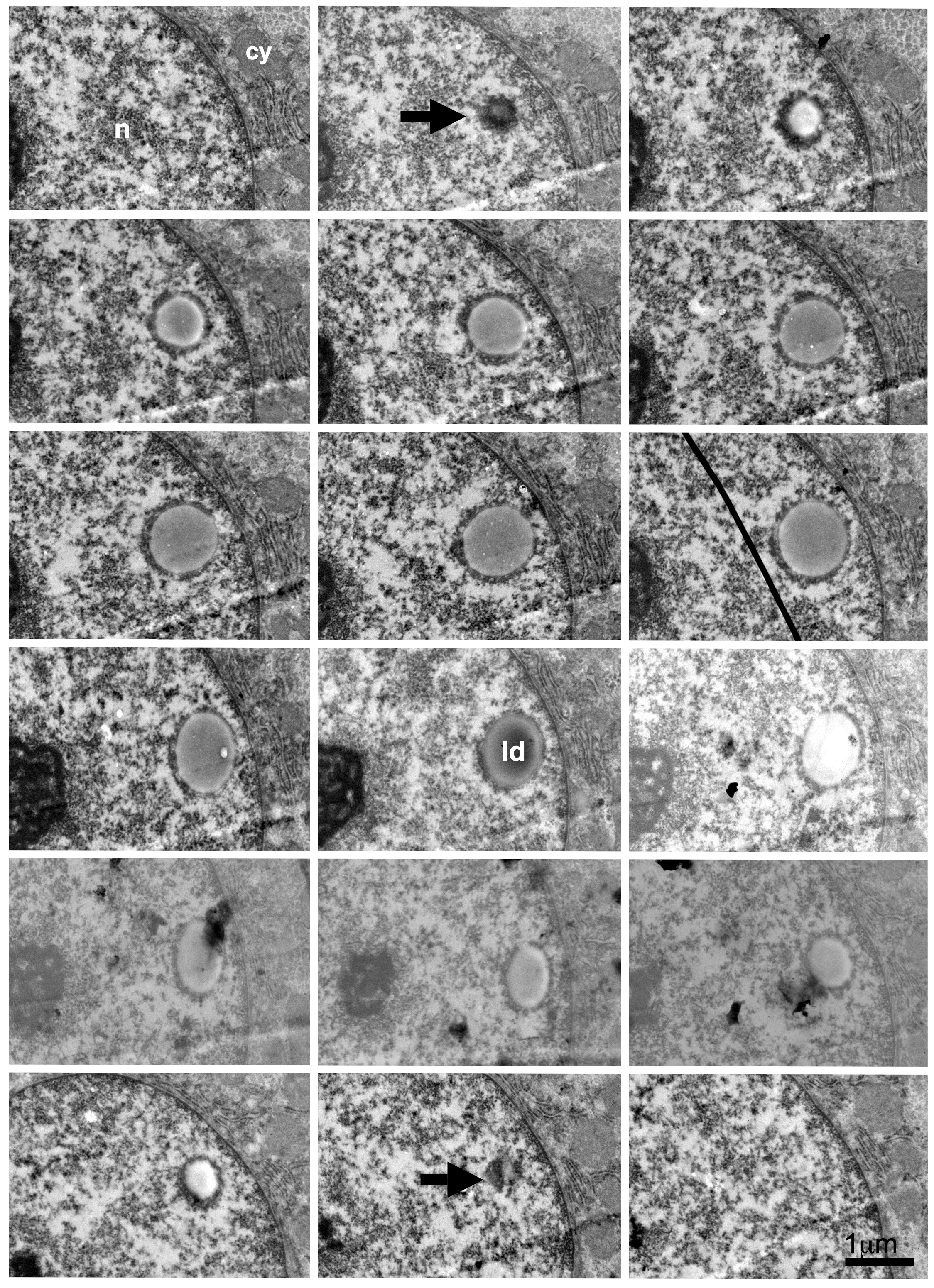

Supplement: Additional file 1: Figure S1 — Serial EM sections of liver tissue showing the presence of a nuclear lipid droplet. These 18 consecutive serial sections show the presence of a nuclear LD, not connected to the nuclear envelope. Arrows indicate the LD in the first and the last sections of the series in which it was possible to visualize the LD. ld: lipid droplet; n: nucleus; cy: cytoplasm. [file 1756-0500-6-386-S1.tiff]
